# Supplementary material for: The interactome of intact mitochondria by cross-linking mass spectrometry provides evidence for coexisting respiratory supercomplexes
Source: Mol Cell Proteomics. 2017 Dec 8;17(2):216–32. doi: 10.1074/mcp.RA117.000470 (PMC5795388; doi:10.1074/mcp.RA117.000470)
Supplement: Supplemental Data [file supp_RA117.000470_133922_0_supp_23978_fzffwf.zip › spectra_annotation/mito_DR_spectra_annotation/169-1-8-1-4-1.pdf]

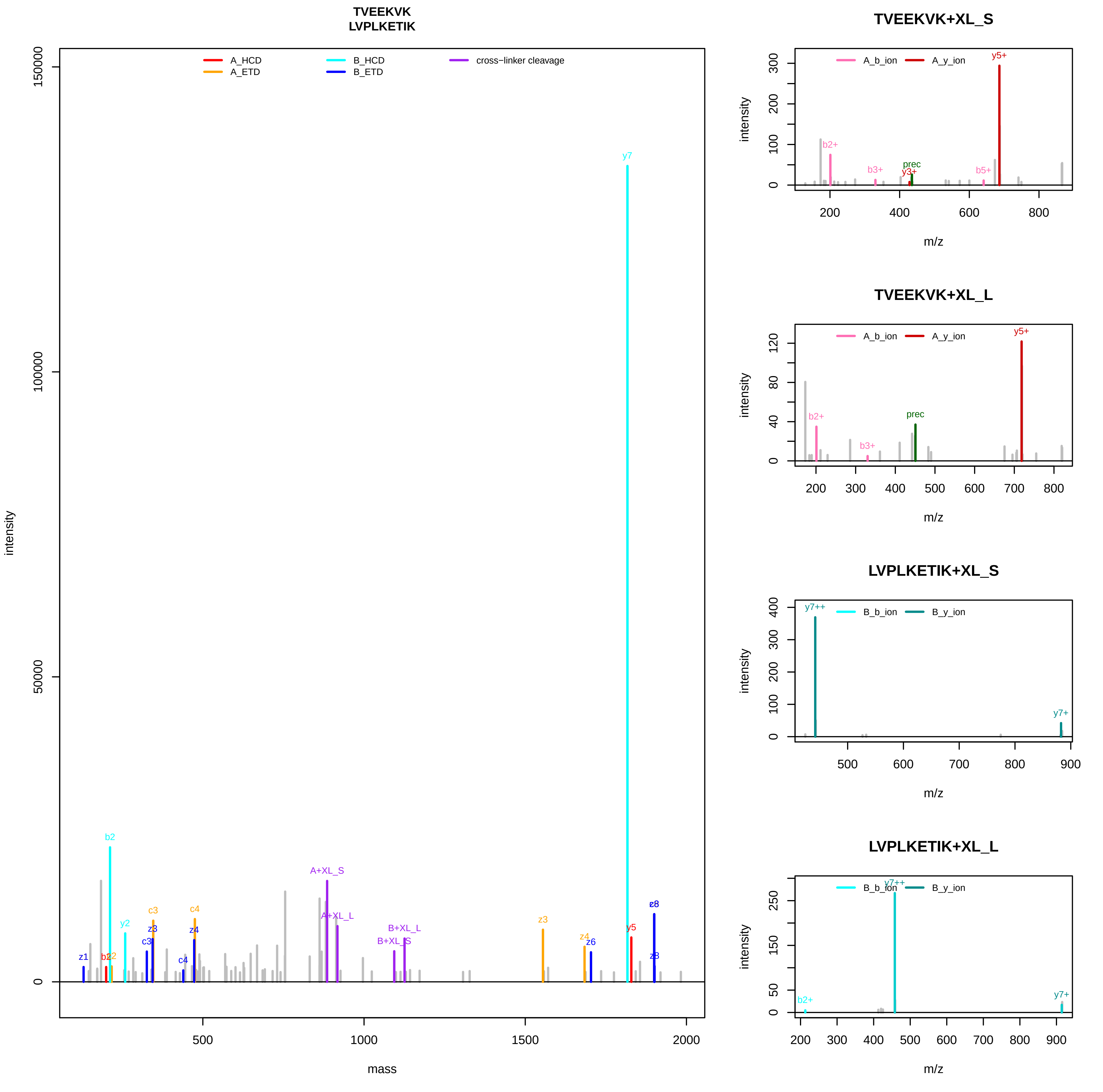

TVEEKVK
LVPLKETIK
A_HCD
A_ETD
B_HCD
B_ETD
cross−linker cleavage
intensity
mass
y7
b2
y2
c3
c4
z3
z4
c3
z1
b2
c4
A+XL_S
A+XL_L
B+XL_L
B+XL_S
z3
z4
z6
y5
z8
TVEEKVK+XL_S
A_b_ion
A_y_ion
y5+
b2+
b3+
prec
y3+
b5+
m/z
TVEEKVK+XL_L
LVPLKETIK+XL_S
B_b_ion
B_y_ion
y7++
y7+
LVPLKETIK+XL_L
b2+
